# Reconstruction Fidelity and Confidence Reliability in Overlapped Radiation-Detector Pulse Analysis Under Distribution Shift

**DOI:** 10.3390/s26206600

**Published:** 2026-10-18

**Authors:** Tao Hong, Bo Zhang, Xinjie Yao, Zhoupeng Guo, Guangsheng Xie

**Affiliations:** 1 China Nuclear Power Engineering Co., Ltd., Beijing 100875, China; 2 CNNC Engineering Research Center for Fuel Reprocessing, Beijing 100875, China; 3 School of Automation Science and Engineering, Xi’an Jiaotong University, Xi’an 710049, China; 4 Faculty of Information Engineering and Automation, Kunming University of Science and Technology, Kunming 650500, China; 5 School of Automation, Southeast University, Nanjing 210096, China; 6 Faculty of Surveying and Information Engineering, West Yunnan University of Applied Sciences, Dali 671000, China

**Keywords:** radiation detection, overlapped pulses, pulse decomposition, confidence calibration, distribution shift, selective prediction

## Abstract

Overlapped radiation-detector pulses must be resolved without conflating accurate pulse counting with reliable recovery of their latent components. This study examines how reconstruction fidelity and predictive confidence respond to controlled changes in detector signals using the public SAEFit dataset and model as a test case. Component reconstruction was assessed using the labeled semi-synthetic protocol, whereas confidence reliability was evaluated under controlled noise and baseline perturbations. Continued training increased pulse-count accuracy to 99.81%, reduced timing RMSE from 2.177 to 1.308 sampling periods, and raised double-pulse ASNR from 20.621 to 21.174 dB. These gains did not extend to amplitude recovery: the amplitude RMSE remained approximately 0.386 compared with the published reference value of 0.056. Under the five principal controlled distribution-shift conditions, the Stage 2 model achieved 60.72% accuracy. Nominally fitted temperature scaling did not improve shifted calibration, whereas condition-matched fitting reduced ECE from 0.3696 to 0.1142 and NLL from 4.5425 to 0.6362, indicating distribution-dependent calibration. These results establish that pulse-count accuracy, component identifiability, and confidence reliability are distinct properties. Calibration improves the interpretation of predictions under the tested perturbations but cannot compensate for poorly identified pulse amplitudes.

## 1. Introduction

Digital radiation-detector systems record waveform traces from which event timing and deposited energy are inferred for nuclear-decay spectroscopy and related nuclear-structure measurements [1,2,3]. As detector arrays and acquisition systems operate at increasing count rates, temporally adjacent events may produce partially overlapped pulses [4,5,6]. Such overlap complicates the extraction of individual pulse parameters and may affect subsequent event identification and spectroscopic interpretation [7]. Reliable and computationally efficient analysis of overlapped waveforms is therefore an important problem in radiation-sensor signal processing.

Recovering the arrival times and amplitudes of constituent pulses from their superposition is an inverse signal-estimation problem. Its difficulty increases as the temporal separation between pulses decreases and waveform noise or baseline variation becomes more pronounced. Conventional pulse-shape analysis generally combines a parametric detector-response model with iterative optimization or least-squares estimation [8,9]. These approaches retain a clear relationship between the assumed response function and the estimated physical parameters, but repeated numerical optimization can become computationally demanding for large waveform datasets. Data-driven estimators provide an alternative by learning the mapping from measured waveforms to pulse multiplicity and component parameters, thereby shifting much of the computational cost from inference to model training [10,11].

SAEFit is a recent example of this approach. It jointly performs pulse-count classification, waveform reconstruction, and response-parameter estimation and uses measured single-pulse waveforms to construct labeled semi-synthetic double-pulse examples [9]. Its public code and data provide a useful basis for examining the behavior of a learned pulse analyzer under a common evaluation protocol. Nevertheless, performance under nominal test conditions does not by itself establish that the resulting predictions remain reliable when the statistical characteristics of the input waveforms change. Variations in noise level and baseline structure can alter both classification performance and the relationship between model confidence and empirical correctness. This issue is particularly relevant when highly confident predictions are used without additional inspection.

In this study, distribution shift refers specifically to a change in the statistical distribution of input waveforms between nominal and evaluation conditions while the prediction task and label semantics remain unchanged. This statistical use of the term should be distinguished from a calibrated physical model of detector drift.

Three aspects of performance must therefore be distinguished. The first is pulse-count classification, which determines whether a waveform contains a single or overlapped event. The second is component-level reconstruction, which concerns the accuracy of the recovered timing and amplitude parameters. The third is confidence reliability, which describes whether the reported class probabilities remain consistent with observed prediction frequencies. These quantities are related but not interchangeable. High pulse-count accuracy does not imply accurate recovery of constituent amplitudes, and improved probability calibration does not correct errors in the reconstructed pulse parameters. Evaluating them separately is necessary to avoid interpreting a strong classification result as evidence of complete waveform decomposition or treating calibrated confidence as evidence of physical reconstruction accuracy.

This distinction is also required by the available ground truth. The public SAEFit release contains measured single-pulse and double-pulse traces, but the measured double-pulse data do not provide independently verified timing and amplitude labels for each constituent pulse. Supervised component-level errors therefore cannot be evaluated on these measured double pulses without introducing additional assumptions or pseudo-labels. Following the original SAEFit protocol, the present study evaluates timing and amplitude recovery using semi-synthetic double pulses generated by shifting, scaling, and summing measured single-pulse waveforms. This construction preserves measured pulse shapes while providing known component parameters, but it does not constitute fully labeled measurement of naturally overlapped events. Accordingly, conclusions concerning component reconstruction are restricted to this semi-synthetic protocol.

Within this evidence boundary, the present study investigates two complementary questions. First, we examine whether continued training and configuration-level adjustment of SAEFit can improve pulse classification and reconstruction while retaining the original data construction, model architecture, and evaluation definitions. Second, we assess the reliability of classification confidence under controlled noise and baseline perturbations. The perturbations are used as reproducible stress tests rather than an exhaustive representation of detector-condition changes. Confidence reliability is evaluated using the expected calibration error (ECE), negative log-likelihood (NLL), the frequency of high-confidence errors, and risk–coverage behavior. Post hoc temperature scaling and confidence-based selective prediction are considered because they modify the interpretation or use of class probabilities without altering the reconstructed waveforms or predicted classes.

The scientific contribution of this study is not a new pulse-analysis architecture or calibration algorithm but an evaluation framework that exposes three distinct properties of an overlapped-pulse analyzer that can otherwise be conflated. First, we establish an explicit evidence boundary between pulse-count classification, component-level reconstruction, and confidence reliability so that conclusions supported by one metric family are not transferred to another. Second, under an unchanged SAEFit architecture and evaluation protocol, we examine whether improvements in pulse-count classification and temporal localization extend to component-amplitude recovery. Five independent repeated runs show statistically supported improvements in classification and timing, whereas the paired changes in component-amplitude RMSE are not statistically significant. Third, under controlled distribution shifts, we examine whether confidence calibration learned under one waveform distribution remains valid under another. The revised calibration analysis shows that post hoc temperature scaling is itself distribution dependent: a scalar temperature estimated from the nominal validation distribution does not necessarily transfer to shifted waveform distributions.

## 2. Related Work

### 2.1. Pulse Analysis and Detector Signal Processing

Digital detector systems have substantially increased the throughput and temporal resolution of nuclear spectroscopy experiments [4,5]. Further optimization of digital acquisition systems for fast scintillator detectors has increased both the volume of recorded waveforms and the demand for efficient waveform processing [6]. Under high-count-rate operation, pulse pile-up and waveform overlap become important sources of uncertainty because a single recorded trace may contain contributions from more than one physical pulse. Pulse-shape analysis is therefore commonly formulated as an inverse estimation problem in which timing, amplitude, and related response parameters are inferred from sampled detector waveforms.

Classical approaches usually rely on response-function modeling and iterative parameter fitting. These methods are attractive because their assumptions are explicit and their estimated parameters can often be interpreted with respect to a physical or instrumental response model. Their limitations are also well known: nonlinear or least-squares fitting can be computationally expensive for large waveform streams, and the resulting estimates may depend on the adequacy of the assumed response model and initialization strategy [9,12]. These limitations have motivated learned waveform analyzers that approximate the mapping from detector traces to pulse classes and pulse parameters more efficiently [8].

SAEFit is a representative recent framework in this direction. It uses a learned pulse-analysis model to classify pulse multiplicity and estimate response parameters for single and double pulses [9]. Its importance for the present study lies not only in the model itself but also the availability of a public dataset and reproducible evaluation protocol. This makes SAEFit a useful basis for examining how reconstruction metrics and reliability metrics should be reported for overlapped radiation-detector pulses. Component-level validation of SAEFit relies on semi-synthetic double pulses because measured double-pulse traces do not contain independently verified constituent timing and amplitude labels. Accordingly, component RMSE values in the present study are interpreted only within this labeled construction protocol.

### 2.2. Neural Estimators and Optimization

Learned estimators have become common in signal-processing tasks where high-dimensional observations must be mapped to task-specific quantities [11]. Residual architectures and attention-based models have shown that learned representations can reduce the need for hand-crafted features in many domains [13,14]. Probabilistic output layers and mixture-based models further provide mechanisms for representing uncertainty or ambiguity in predicted targets [15,16]. For detector-pulse analysis, however, model capacity alone is not sufficient. Since timing and amplitude estimates may enter downstream physical interpretation, improvements in learned representations must be evaluated against clearly defined measurement targets and label sources.

The present work does not introduce a new pulse model, architecture, loss function, or optimizer. Instead, SAEFit is used as a controlled test case to examine a different question: whether improvements in pulse-count classification and waveform reconstruction imply reliable recovery of latent pulse parameters and whether classification confidence remains statistically interpretable when the input waveform is perturbed. The contribution is therefore an evaluation of the consistency among task performance, physical reconstruction fidelity, and confidence reliability under fixed model semantics.

The optimization procedures used in this setting are standard components of modern neural training. Adam-style optimizers, decoupled weight decay, and widely used PyTorch-based implementations (PyTorch version 2.8.0) provide the computational infrastructure for continuation experiments [17,18,19,20]. We therefore treat optimization as a controlled experimental procedure rather than the primary methodological contribution. This distinction is important for reproducibility because the scientific claim depends on whether reconstruction and calibration results are obtained under fixed evaluation semantics rather than the novelty of the optimizer or implementation stack.

### 2.3. Uncertainty, Calibration, and Abstention

Uncertainty estimation and calibration are closely related but distinct aspects of reliable prediction. Predictive uncertainty can be approximated using methods such as Bayesian dropout, deep ensembles, and heteroscedastic regression [21,22,23,24]. Post hoc calibration methods provide a complementary approach by adjusting predictive confidence after model training without necessarily changing the predicted class or regression output [25,26,27]. These methods are particularly relevant when a model is already trained and the main question is whether its reported confidence is aligned with observed correctness.

The statistical basis of calibration predates modern neural models. Well-calibrated probabilistic prediction has been studied from Bayesian and frequentist perspectives [28], and probability-score decomposition provides a principled way to separate different aspects of forecast quality [29]. Reliability diagrams and related calibration diagnostics remain useful tools for assessing whether predicted confidence values correspond to empirical accuracy [30]. In sensor applications, this distinction is important because a prediction may be numerically accurate under nominal conditions while becoming overconfident under shifted operating conditions.

Distribution shift is a central challenge for learned sensor systems. Changes in noise level, baseline behavior, environmental conditions, or acquisition electronics can alter the input distribution encountered during deployment. Prior studies have shown that such variability can degrade model robustness and confidence reliability [31,32]. Recent sensor-oriented work has therefore considered uncertainty-aware or distribution-aware decision mechanisms under changing conditions [33]. For radiation-detector pulse analysis, the same concern arises when a model that was trained or tuned under one waveform condition is applied to traces with different noise or baseline characteristics.

Reliable confidence estimates can also support abstention. Selective classification formalizes the trade-off between coverage and risk by allowing a model to withhold predictions on examples that are determined to be insufficiently reliable [34,35]. Conformal prediction provides a related framework for distribution-free uncertainty quantification under appropriate assumptions [36,37], and recent sensor studies have used conformal calibration to construct reliable prediction intervals [38]. In the present study, selective prediction is used more modestly as a risk–coverage diagnostic rather than a formal conformal guarantee.

Taken together, these studies motivate a cautious evaluation strategy. Calibration and abstention can improve how confidence is reported and used, but they do not repair inaccurate physical decomposition. A detector-pulse analyzer that performs well on clean validation traces may still assign excessive confidence to shifted or ambiguous traces, while a calibrated confidence score does not prove that subpulse amplitudes have been accurately recovered. The present work therefore treats uncertainty calibration as a decision layer on top of a fixed decomposition model and reports it separately from component-level reconstruction performance.

## 3. Methods

### 3.1. Study Design and Evidence Boundary

The study was designed to evaluate two distinct properties of the SAEFit pulse analyzer: component-reconstruction fidelity under the original evaluation protocol and classification-confidence reliability under controlled signal perturbations. These properties were assessed separately because the available reference information differs between the two settings and confidence calibration does not alter the reconstructed pulse parameters.

For a normalized detector waveform x, SAEFit produces class logits z, a reconstructed waveform $\hat{x}$, and estimated response parameters describing pulse timing, amplitude, and shape [9]. The classifier distinguishes single- from double-pulse traces, whereas the reconstruction and parameter-estimation outputs are used to evaluate waveform and component-level fidelity.

Measured double-pulse traces lack independently verified component timing and amplitude labels. Accordingly, supervised component-level errors are evaluated only on semi-synthetic double pulses constructed from measured single pulses by temporal shifting, amplitude scaling, and summation according to the SAEFit protocol, thereby providing known component timing and amplitude values. Measured double-pulse traces are retained only as evidence of naturally occurring overlap in the public dataset and are not used for supervised component-level performance claims. Table 1 summarizes the resulting evidence boundary.

**Figure 1:**
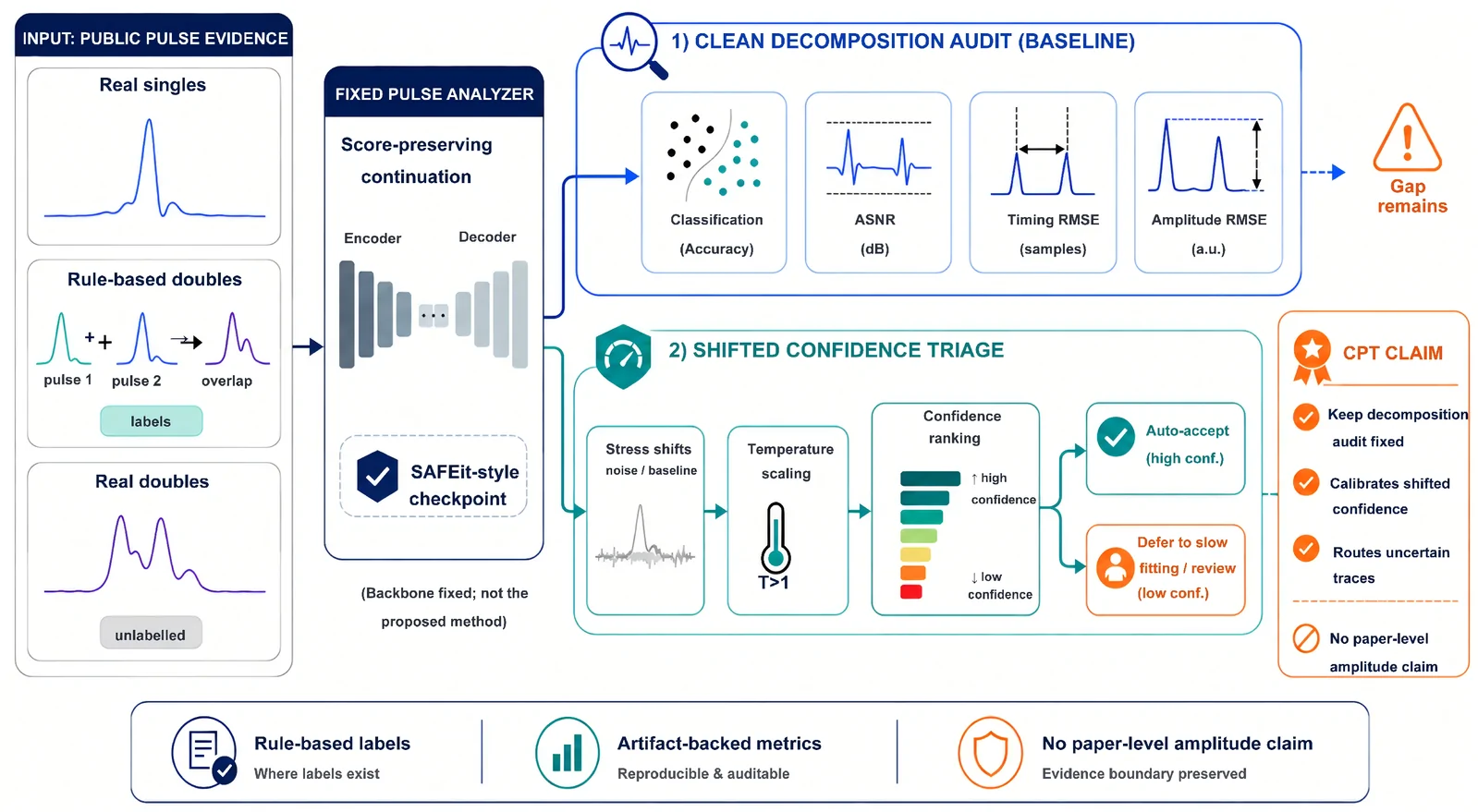
Overview of the evaluation design. Pulse classification and component reconstruction are assessed using the unchanged SAEFit data protocol, whereas confidence reliability is evaluated separately under controlled noise and baseline perturbations. Post hoc calibration and confidence-based selection operate only on classification probabilities and do not modify the reconstructed waveforms or estimated pulse parameters.

Accordingly, timing and amplitude RMSE are reported only for the labeled semi-synthetic protocol. Controlled noise and baseline perturbations are used only to examine pulse-count classification and confidence behavior. They are not used to infer component-level reconstruction errors. Figure 1 summarizes this evaluation design.

### 3.2. Protocol-Preserving Model Continuation

The continuation experiments examine whether additional training and adjustment of existing optimization controls improve SAEFit performance without changing the underlying problem definition. The model architecture, waveform-construction procedure, data split, target labels, and metric implementations are retained from the public SAEFit protocol. The varied quantities are restricted to training controls that are already supported by the implementation: learning rate, continuation duration, checkpoint selection, the timing-loss weight τ, the classification-loss weight $\tau_{c}$, and the reconstruction-related weight λ.

Continuation is performed in two stages. The first stage prioritizes temporal localization by increasing the contribution of the timing objective while reducing the weight assigned to the already high-performing pulse-count classifier. Timing RMSE is treated as the primary quantity for checkpoint comparison in this stage, while classification accuracy and waveform-reconstruction metrics are monitored for deterioration. The second stage examines whether adjustment of the reconstruction-related loss weight improves component-amplitude estimation without substantially degrading the other reported metrics. Amplitude RMSE is treated as the primary quantity in this stage.

This procedure is not introduced as a new learning algorithm. It is a controlled examination of the extent to which the performance of an existing SAEFit model can change under additional training and configuration-level adjustment. All the comparisons use the same evaluation definitions, and results are reported for the full metric set rather than only the metric prioritized during a given stage. This prevents an improvement in timing or classification from being interpreted as a general improvement in component reconstruction.

### 3.3. Confidence Reliability Under Controlled Signal Perturbations

Confidence reliability is evaluated using perturbed versions of normalized evaluation traces. Let $x_{i}$ denote an unperturbed normalized waveform. A perturbed waveform is defined as

(1) $$x_{i}^{\left(s\right)}=x_{i}+\eta_{i}^{\left(s\right)}+b_{i}^{\left(s\right)},$$

where $\eta_{i}^{\left(s\right)}$ denotes additive waveform noise, $b_{i}^{\left(s\right)}$ denotes a baseline perturbation, and *s* identifies the perturbation condition. The perturbations are applied after the original waveform normalization and before model inference. Five conditions are considered: noise only, baseline only, a reference combined condition, a mild combined condition, and a strong combined condition. The corresponding perturbation magnitudes and sampling procedures are specified in the experimental protocol.

The original pulse-count label is retained because the perturbations are assumed not to change whether the underlying waveform contains one or two pulses. This assumption defines the scope of the stress test. The perturbed traces represent controlled departures from the nominal evaluation distribution; they are not claimed to reproduce the full range of changes that may occur in detector response, electronics, sampling, or experimental conditions.

For each perturbed waveform, the model produces logits $z_{i}$. The probability assigned to class *k* is

(2) $$p_{ik}=\frac{\exp(z_{ik})}{\sum_{j=1}^{K}\exp\left(z_{ij}\right)},$$

where K=2 for single- and double-pulse classification. The predicted class and associated confidence are

(3) $${\hat{y}}_{i}=\underset{k}{\arg\max}\;p_{ik},\;c_{i}=\max_{k}p_{ik}.$$

No perturbed trace is used to calculate component timing or amplitude RMSE.

### 3.4. Reconstruction and Classification Metrics

Let $\delta_{{\hat{y}}_{i},y_{i}}$ denote the Kronecker delta, which equals 1 when ${\hat{y}}_{i}=y_{i}$ and 0 otherwise. Pulse-count accuracy is calculated as

(4) $$\mathrm{Acc}=\frac{1}{n}\sum_{i=1}^{n}\delta_{{\hat{y}}_{i},y_{i}},$$

where $y_{i}$ is the reference pulse-count label.

Following the public SAEFit evaluation convention, waveform-reconstruction quality is summarized using the average signal-to-noise ratio (ASNR). Let $s_{ij}$ and ${\hat{s}}_{ij}$ denote the target and reconstructed values, respectively, at sample *j* of trace *i*. The trace-level signal-to-noise ratio is

(5) $${\mathrm{SNR}}_{i}=10\log_{10}\left[\frac{\sum_{j=1}^{L}s_{ij}^{2}}{\sum_{j=1}^{L}{\left(s_{ij}-{\hat{s}}_{ij}\right)}^{2}}\right],$$

where *L* is the number of waveform samples. ASNR is the mean over all evaluated traces:

(6) $$\mathrm{ASNR}=\frac{1}{n}\sum_{i=1}^{n}{\mathrm{SNR}}_{i}.$$

ASNR is reported in decibels, with larger values indicating closer waveform agreement. Separate values are reported for single pulses, individual subpulses, and reconstructed double pulses when the corresponding reference waveform is available.

For a component-level target $q_{i}$ and its estimate ${\hat{q}}_{i}$, RMSE is defined as

(7) $$\mathrm{RMSE}\left(q,\hat{q}\right)=\sqrt{\frac{1}{n}\sum_{i=1}^{n}{\left(q_{i}-{\hat{q}}_{i}\right)}^{2}}.$$

Timing RMSE is expressed in sampling periods, whereas amplitude RMSE is calculated using normalized component amplitudes. For double pulses, amplitude errors are reported separately for the two ordered components. Because these quantities require component labels, they are evaluated only on semi-synthetic double pulses. For double pulses, we additionally define the joint component-amplitude RMSE as

(8) $${\mathrm{RMSE}}_{a,\mathrm{joint}}=\sqrt{\frac{1}{2N}\sum_{i=1}^{N}\left[{\left(a_{i,1}-{\hat{a}}_{i,1}\right)}^{2}+{\left(a_{i,2}-{\hat{a}}_{i,2}\right)}^{2}\right]}.$$

This metric summarizes the amplitude-reconstruction error of both ordered components within a single quantity.

### 3.5. Confidence-Calibration Metrics

Confidence reliability under controlled perturbation is assessed using the expected calibration error (ECE), negative log-likelihood (NLL), and overconfident error rate. For ECE, predictions are partitioned into *B* confidence intervals, denoted by $I_{1},\ldots ,I_{B}$. The empirical accuracy of bin $I_{b}$ is

(9) $$\mathrm{acc}\left(I_{b}\right)=\frac{1}{|I_{b}|}\sum_{i\in I_{b}}\delta_{{\hat{y}}_{i},y_{i}},$$

and its mean confidence is

(10) $$\mathrm{conf}\left(I_{b}\right)=\frac{1}{|I_{b}|}\sum_{i\in I_{b}}c_{i}.$$

ECE is calculated as

(11) $$\mathrm{ECE}=\sum_{b=1}^{B}\frac{|I_{b}|}{n}\left|\mathrm{acc}\left(I_{b}\right)-\mathrm{conf}\left(I_{b}\right)\right|.$$

Because ECE depends on the binning scheme, the number and definition of the confidence bins are held fixed for all comparisons.

NLL is evaluated directly from the classification logits as

(12) $$\mathrm{NLL}=-\frac{1}{n}\sum_{i=1}^{n}\mathrm{logsoftmax}{\left(z_{i}\right)}_{y_{i}}.$$

For temperature-scaled predictions, $z_{i}$ is replaced by $z_{i}/T^{*}$. Unlike classification accuracy, NLL depends on the probability assigned to the reference class and therefore strongly penalizes confident errors. In the implementation, NLL is computed using the numerically stable log-softmax/cross-entropy formulation rather than by explicitly taking the logarithm of softmax probabilities. This avoids numerical underflow when the probability assigned to the reference class is very small.

For a confidence threshold ρ, let

(13) $$S_{\rho }=\left\{i:c_{i}\geq \rho \right\}$$

denote the subset of predictions whose confidence is at least ρ. We report two complementary definitions of overconfident error. The conditional overconfident error rate is

(14) $${\mathrm{OER}}_{\mathrm{cond}}\left(\rho \right)=\frac{\sum_{i=1}^{n}I\left({\hat{y}}_{i}\neq y_{i},\;c_{i}\geq \rho \right)}{\sum_{i=1}^{n}I\left(c_{i}\geq \rho \right)},$$

provided that at least one prediction satisfies the confidence threshold. If no prediction satisfies the threshold, ${\mathrm{OER}}_{\mathrm{cond}}$ is treated as undefined.

The absolute overconfident error rate is

(15) $${\mathrm{OER}}_{\mathrm{abs}}\left(\rho \right)=\frac{1}{n}\sum_{i=1}^{n}I\left({\hat{y}}_{i}\neq y_{i},\;c_{i}\geq \rho \right).$$

The conditional definition measures the error probability among retained high-confidence predictions, whereas the absolute definition measures the frequency of high-confidence errors relative to the complete evaluation set.

For completeness, threshold-dependent coverage is defined as

(16) $$\mathrm{Coverage}\left(\rho \right)=\frac{1}{n}\sum_{i=1}^{n}I\left(c_{i}\geq \rho \right).$$

Rather than treating ρ=0.85 as a uniquely justified operating threshold, we retain it only as a reference diagnostic point. Threshold sensitivity is evaluated over

ρ∈{0.50,0.60,0.70,0.75,0.80,0.85,0.90,0.95,0.99,0.995,0.999,0.9999}.

### 3.6. Post Hoc Temperature Scaling

Temperature scaling is applied to the classification logits using a scalar parameter T>0 [27]. The scalar temperature is estimated exclusively from validation logits by minimizing validation negative log-likelihood. The revised implementation first evaluates a logarithmically spaced set of candidate temperatures and then applies golden-section refinement. No evaluation labels are used during temperature fitting.

(17) $$T^{*}=\underset{T>0}{\arg\min}\left\{-\frac{1}{n_{\mathrm{val}}}\sum_{i=1}^{n_{\mathrm{val}}}\log\left[\frac{\exp(z_{i,y_{i}}/T)}{\sum_{k=1}^{K}\exp\left(z_{ik}/T\right)}\right]\right\}.$$

The calibrated probability assigned to class *k* for a held-out evaluation trace is

(18) $$p_{ik}^{\left(T\right)}=\frac{\exp(z_{ik}/T^{*})}{\sum_{j=1}^{K}\exp\left(z_{ij}/T^{*}\right)}.$$

For $T^{*}>0$, temperature scaling preserves the ordering of the logits and therefore does not change the predicted class. Differences between calibrated and uncalibrated results are consequently restricted to confidence-dependent quantities such as ECE, NLL, the overconfident error rate, and selective risk. Temperature scaling does not modify waveform reconstruction or component-parameter estimates.

### 3.7. Selective Prediction

Selective prediction is evaluated by ranking traces according to their maximum calibrated confidence:

(19) $$c_{i}^{\left(T\right)}=\max_{k}p_{ik}^{\left(T\right)}.$$

For target coverage γ, let $A_{\gamma }$ contain the ⌈γn⌉ traces with the largest calibrated confidence values. Selective accuracy is

(20) $${\mathrm{Acc}}_{\gamma }=\frac{1}{|A_{\gamma }|}\sum_{i\in A_{\gamma }}\delta_{{\hat{y}}_{i},y_{i}},$$

and selective risk is

(21) $${\mathrm{Risk}}_{\gamma }=1-{\mathrm{Acc}}_{\gamma }.$$

The resulting risk–coverage curve assesses whether confidence ranking identifies subsets with different empirical error rates.

This procedure is a selective-classification diagnostic and is not a conformal-prediction method. It does not construct prediction sets, establish finite-sample coverage, or provide a distribution-free guarantee. The reported coverage denotes the proportion of traces retained after confidence ranking rather than a guaranteed probability of containing a correct label.

### 3.8. Protocol Control and Reproducibility

All the nominal reconstruction comparisons retain the public SAEFit data split, semi-synthetic waveform-construction rules, label definitions, and metric implementations. Training configurations and selected checkpoints are recorded for each continuation experiment. Calibration parameters are estimated using validation predictions and evaluated using held-out predictions. The uncalibrated and calibrated metrics are computed from the same logits so that temperature scaling cannot change the underlying classification decisions.

Results that do not support the intended hypothesis are retained in the analysis. In particular, component-amplitude RMSE is reported alongside timing and waveform metrics. The absence of component labels for measured double pulses is handled by restricting the corresponding conclusions rather than introducing pseudo-labels. These controls ensure that reported improvements are attributable to the specified training or calibration operation and do not result from changes in the evaluation definition.

## 4. Experimental Protocol

### 4.1. Data Use and Reference Models

All the experiments were conducted using the public SAEFit code and data release [9]. The original waveform preprocessing, data partition, semi-synthetic pulse-construction procedure, and metric implementations were retained. No additional detector dataset or independently generated component label was introduced.

The public data were used according to the availability of reference labels. Measured single-pulse traces were used directly and also shifted, amplitude-scaled, and summed according to the public SAEFit procedure to construct labeled semi-synthetic double pulses. These semi-synthetic traces provide known component delays and amplitudes and therefore support supervised timing and amplitude evaluation. The measured double-pulse traces do not contain independently verified labels for the constituent timing and amplitude parameters. They were consequently excluded from supervised component-level RMSE calculations.

The semi-synthetic construction contains 160 valid timing–amplitude configurations. For each valid configuration, 3000, 350, and 700 double-pulse examples are generated for training, validation, and evaluation, respectively, yielding 480,000 training, 56,000 validation, and 112,000 evaluation semi-synthetic double pulses. For the revised classification and calibration analyses, the 112,000 double-pulse evaluation examples are combined with an equal number of single-pulse instances. Each evaluation condition therefore contains 224,000 traces with a balanced 1:1 single-/double-pulse class composition.

The semi-synthetic double pulses follow the public SAEFit construction procedure. The measured single-pulse pool is first partitioned into training, validation, and evaluation subsets using the public 70%/10%/20% split with random seed 0. The measured single-pulse pool contains 171,230 usable 250-sample traces after application of the public SAEFit loader’s saturation rule, corresponding to 119,860 training, 17,124 validation, and 34,246 evaluation traces. For each generated example, both components are derived from the same measured single-pulse waveform rather than two independently selected pulses. The first-component temporal shift is sampled from four integer intervals, [−84,−42), [−42,0), [0,42), and [42,84) samples, while the relative delay of the second component is sampled from [2,31), [31,61), [61,91), [91,121), [121,151), and [151,176) samples. Relative amplitude scaling is sampled log-uniformly within ten intervals spanning 0.1–10: [0.1,0.125], [0.125,1/6], [1/6,0.25], [0.25,0.5], [0.5,1], [1,2], [2,4], [4,6], [6,8], and [8,10]. Temporal shifting is implemented at integer-sample resolution using the NumPy roll operation; samples vacated by the shift are filled using the corresponding waveform edge value to prevent circular wrap-around. Saturated source pulses are excluded, and timing configurations are retained only when they satisfy the maximum-position constraint of the public SAEFit implementation.

Three model-reference levels were distinguished. The first was the locally reproduced epoch-125 SAEFit checkpoint, which provided the initial reference for the continuation experiments. It achieved a pulse-count accuracy of 99.40%, a single-pulse ASNR of 21.12 dB, subpulse ASNR values of 16.76 and 16.89 dB, and a double-pulse ASNR of 20.06 dB. The second reference consisted of the values reported for the 750-epoch model in the original SAEFit study: 99.63% pulse-count accuracy, 21.57 dB single-pulse ASNR, 20.20 and 20.32 dB subpulse ASNR, 21.94 dB double-pulse ASNR, a timing RMSE of 0.71 sampling periods, and amplitude RMSE values of 0.058 and 0.056. These published values were used only for contextual comparison and were not re-estimated or used as training targets. The third reference was the final checkpoint selected from the continuation experiments described below.

### 4.2. Continuation Stages and Repeated-Run Protocol

The epoch-125 checkpoint was first continued toward approximately 500 training epochs while retaining the original model architecture and evaluation procedure. A checkpoint near the end of this continuation stage was then used to initialize two sequential configuration stages. The first stage prioritized timing estimation, whereas the second examined whether reconstruction-related weighting could improve component-amplitude estimation.

The selected configurations are summarized in Table 2. Apart from the listed parameters, the optimizer, scheduler, data construction, and remaining training settings were retained from the public implementation.

The timing-focused stage increased the contribution of the timing objective while reducing the weight assigned to the already high-performing classifier. Checkpoint comparison primarily considered timing RMSE, while classification accuracy and ASNR were monitored to identify possible deterioration in other outputs. The amplitude-focused stage started from the selected timing-focused checkpoint and increased the reconstruction-related weight. Amplitude RMSE was treated as the primary evaluation quantity, with timing, classification, and ASNR retained as complementary metrics.

The configuration search was deliberately bounded and was not intended as an exhaustive hyperparameter optimization. Because loss-weight adjustment and checkpoint selection used feedback from the public SAEFit evaluation framework, the selected checkpoint metrics reported here are protocol-optimized results rather than untouched estimates from an independent test set. This distinction is maintained throughout the Results and Discussion. Both continuation stages used a fixed 100-epoch training budget. Validation was performed every 10 epochs for Stage 1 and every 50 epochs for Stage 2. Early stopping was disabled, and the retained checkpoints were selected from the scheduled validation/checkpoint records within the fixed training budget.

To quantify run-to-run variability, both continuation stages were independently repeated using five random seeds, {0,1,2,3,4}. The model architecture, data protocol, loss weights, learning rates, and training budgets were held fixed across seeds. The same seed was paired between the timing-focused Stage 1 and the amplitude-focused Stage 2. Principal metrics are reported as mean ± standard deviation together with paired 95% confidence intervals for the Stage 2–Stage 1 difference.

### 4.3. Controlled Signal-Perturbation Protocol

Confidence reliability was assessed using controlled perturbations applied to normalized evaluation traces. The perturbations were introduced after waveform normalization and before model inference. The pulse-count labels were retained because the perturbations were designed to modify signal quality without changing whether the underlying trace contained one or two pulses.

Five perturbation conditions were considered:

1. Additive waveform noise only;
2. Baseline variation only;
3. A reference combined noise-and-baseline condition;
4. A mild combined condition;
5. A strong combined condition.

For the additive-noise conditions, independent zero-mean Gaussian noise was added to each normalized waveform. Baseline perturbation was implemented as a trace-wise constant offset sampled from a uniform distribution. The numerical settings are summarized in Table 3.

No additional colored-noise spectrum was imposed. These five settings were designed as reproducible signal-level stress tests rather than calibrated models of the operating statistics of a particular detector system.

Two additional controlled transformation families were included as supplementary distribution-shift stress tests. First, multiplicative gain factors

g∈{0.80,0.90,0.95,1.00,1.05,1.10,1.20}

were used to examine sensitivity to gain variation. Second, 16 parametric pulse-shape conditions were evaluated to examine sensitivity to waveform-shape changes. These supplementary tests broaden the controlled waveform variations considered in this study but are not intended as a complete physical model of detector-condition variation. These transformations are used as controlled input-distribution stress tests and are not intended as a calibrated simulator of the physical drift of a specific detector or acquisition system.

The same final continued checkpoint was evaluated under every condition. Perturbed traces were not used to retrain the decomposition model and were not used to calculate component timing or amplitude RMSE. The perturbation experiments were restricted to pulse-count accuracy and confidence-dependent metrics, including ECE, NLL, conditional and absolute overconfident error rates, and selective risk. OER threshold sensitivity was evaluated over the predefined threshold set in Section 3.5, with ρ=0.85 retained only as a reference diagnostic point.

The resulting observations are therefore restricted to the perturbation types and intensities evaluated in this controlled stress test and should not be interpreted as evidence of robustness to general detector-condition changes.

### 4.4. Confidence Calibration and Selective Prediction

For each perturbation condition, uncalibrated class probabilities were obtained from the softmax outputs of the selected checkpoint. Temperature scaling was fitted using the corresponding validation logits by minimizing NLL. In the primary calibration-transfer protocol, temperature was fitted on the nominal validation distribution. The resulting temperatures were T=1.028994 for Stage 1 and T=0.972805 for Stage 2. A separate condition-matched sensitivity analysis fitted temperature using validation data generated under the reference combined perturbation. This analysis is reported separately and is not interpreted as zero-shot calibration transfer. This scalar was subsequently applied to the held-out evaluation logits.

Because division by a positive temperature preserves the ordering of class logits, temperature scaling does not change the predicted pulse-count class. Calibrated and uncalibrated accuracy must therefore be identical when they are calculated from the same logits. The comparison was restricted to confidence-sensitive quantities, namely ECE, NLL, the overconfident error rate, and risk–coverage behavior.

Selective prediction was evaluated by ranking traces according to maximum temperature- scaled confidence. For each target coverage, the highest-confidence fraction of traces was retained, and selective accuracy and selective risk were calculated for that subset. Coverage therefore denotes the empirical proportion of retained traces. This experiment provides a risk–coverage diagnostic and does not constitute conformal prediction or provide a distribution-free coverage guarantee.

### 4.5. Comparison Strategy

The three reference levels address different experimental questions and were therefore not combined into a single aggregate score. Comparison with the epoch-125 checkpoint assesses whether continued training and configuration adjustment improve the local SAEFit reproduction. Comparison with the published 750-epoch values is used only to provide contextual scale for the local results and is not interpreted as a controlled performance comparison. The published and locally continued models originate from different training histories, and no paired statistical inference is made between them.

The local epoch-125 checkpoint and the published 750-epoch reference originate from different training histories. Their comparison is therefore descriptive rather than a paired statistical comparison. Similarly, exceeding a published value for one metric does not establish general superiority when other reconstruction quantities remain below the published reference. Classification accuracy, ASNR, timing RMSE, amplitude RMSE, and calibration metrics are consequently reported separately.

The uncertainty analysis is also interpreted independently of the nominal reconstruction comparison. A reduction in ECE or NLL does not imply improved component timing or amplitude estimation, and an unchanged shifted accuracy does not invalidate a calibration improvement. The two analyses address different properties of the same checkpoint.

### 4.6. Implementation and Reproducibility Controls

The continuation experiments used the public SAEFit training stack and retained the original model interface, optimizer family, scheduler, waveform-construction procedure, and metric implementations. No additional neural module was introduced. Configuration changes were restricted to the parameters reported in Table 2.

For calibration, the trained checkpoint was loaded once and the classification logits were recorded for the validation and evaluation partitions. The same evaluation logits were used for calibrated and uncalibrated comparisons. This paired construction ensures that any difference in confidence-sensitive metrics results from probability rescaling rather than different input traces or classification decisions.

Quantitative figures were generated programmatically from the recorded numerical results. For the target-normalized reconstruction plot presented in the Section 5, higher-is-better quantities were normalized as the observed value divided by the published reference value. Lower-is-better quantities were normalized as the published reference value divided by the observed value. A normalized value of one therefore indicates equality with the published reference for either type of metric. Exact unnormalized values are reported in the corresponding tables and should be used for quantitative interpretation.

All the reported reconstruction metrics were calculated using unchanged target definitions. Unfavorable results were retained when they constrained the interpretation of the model. In particular, amplitude RMSE was reported alongside timing and ASNR, and the absence of component labels for measured double pulses was handled by restricting the evaluation scope rather than introducing pseudo-labels. These controls prevent calibration improvements from being interpreted as evidence of improved pulse decomposition.

## 5. Results

### 5.1. Reconstruction Performance Under the Nominal Protocol

Table 4 reports pulse-count classification, waveform reconstruction, timing estimation, and second-component-amplitude estimation under the nominal SAEFit evaluation protocol. The published 750-epoch values are included as contextual references rather than reproduced results in the present study.

Relative to the continued checkpoint, timing-focused continuation reduced timing RMSE from 2.177 to 1.402 sampling periods. It also increased the first- and second-component ASNR values from 18.59 and 18.16 dB to 19.17 and 18.79 dB, respectively, and increased double-pulse ASNR from 20.62 to 21.10 dB. The subsequent amplitude-focused stage further reduced timing RMSE to 1.308 sampling periods and increased double-pulse ASNR to 21.17 dB. Pulse-count accuracy changed only slightly across these stages, from 99.77% to 99.81%.

Because the archived nominal-protocol records did not contain the complete component-amplitude metric set for every checkpoint, the two final continuation stages were additionally evaluated using a common component-level evaluator on the same 112,000 semi-synthetic double-pulse examples. The resulting component-specific and joint amplitude errors are reported separately in Table 5 to avoid mixing metrics obtained from different evaluation passes.

The complete component-level reevaluation shows essentially no improvement in amplitude recovery between the two continuation stages. RMSE_*a*,1_ changed from 1.5511 to 1.5518, RMSE_*a*,2_ remained approximately 0.3807, and RMSE_*a*,joint_ changed from 1.1293 to 1.1298. These small differences indicate that the amplitude-focused continuation did not materially improve component-amplitude recovery.

In contrast to the clear changes in timing RMSE and waveform ASNR, component-amplitude recovery remained effectively unchanged within the tested configuration range. The second-component-amplitude RMSE decreased only from 0.3869 to 0.3859, corresponding to a relative change of approximately 0.26%. This difference is too small to support a meaningful claim of improved amplitude recovery, particularly when training variability is taken into account. More importantly, the final value remained approximately 6.9 times larger than the published reference value of 0.0560. Because pulse amplitude is directly related to the energy information carried by detector events, this residual error represents a physically important limitation rather than a minor auxiliary metric. The repeated-run analysis changes the interpretation of this single-checkpoint difference. Across five independent seeds, Stage 2 produces statistically supported improvements in pulse-count accuracy and timing RMSE, whereas the paired 95% confidence intervals for changes in both component-amplitude RMSE values include zero. We therefore do not interpret the small single-run amplitude reduction as a statistically supported improvement in component-amplitude recovery.

To determine whether these checkpoint-level differences were reproducible across training trajectories, we repeated the two continuation stages using five independent random seeds. The results are summarized in Table 6. Across the five runs, Stage 2 increased mean pulse-count accuracy from (98.85±0.1250)% to (99.21±0.0906)% and reduced timing RMSE from 2.4776±0.1773 to 2.1849±0.2196 sampling periods. The paired 95% confidence intervals for both changes excluded zero.

In contrast, the paired confidence intervals for the changes in first- and second-component-amplitude RMSE both included zero. The repeated-run results therefore support systematic improvements in pulse-count classification and timing estimation but do not provide statistical evidence for improved component-amplitude recovery. Here, Stage 1 denotes the timing-focused continuation and Stage 2 denotes the subsequent amplitude-focused continuation.

**Figure 2:**
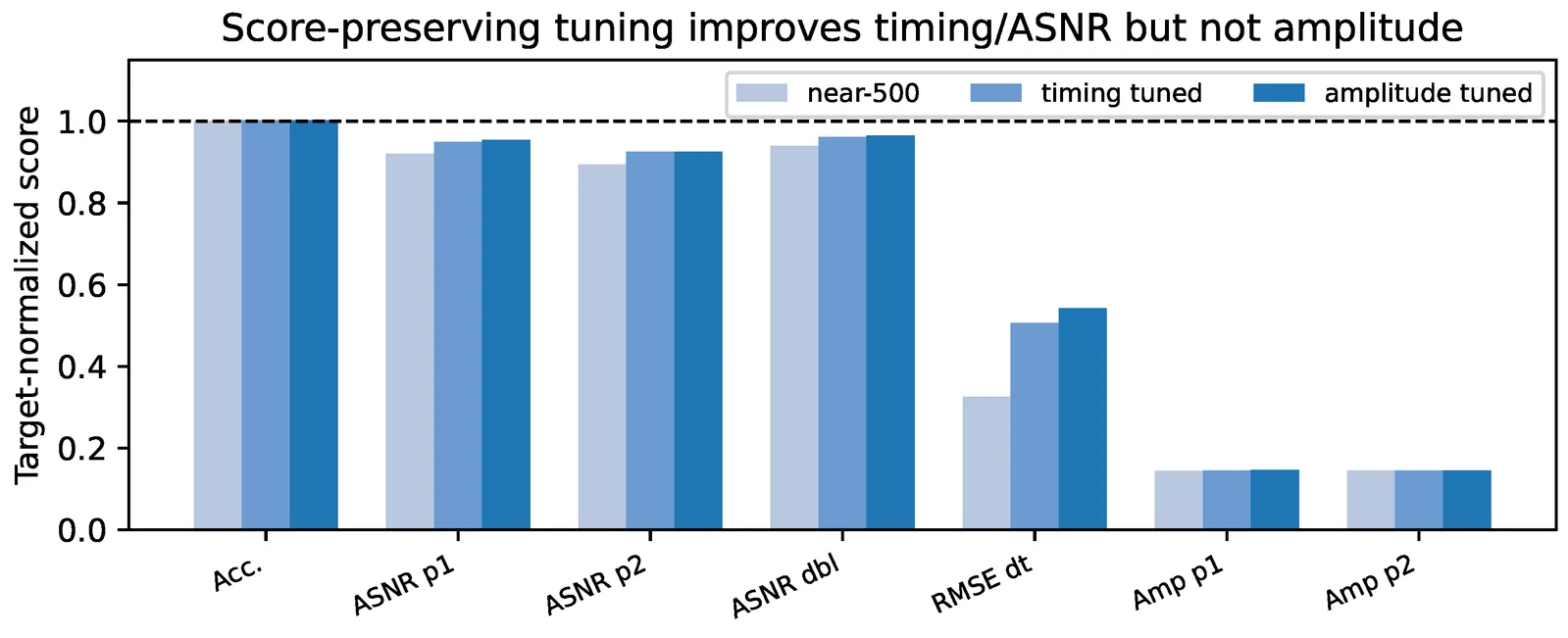
Target-normalized reconstruction performance of the continued checkpoints. Higher-is-better and lower-is-better metrics are normalized relative to the published reference values in opposite directions.

Figure 2 presents the same comparison after normalization by the published reference values. For higher-is-better metrics, the observed value is divided by the published reference value. For lower-is-better metrics, the published reference value is divided by the observed value. Values approaching one indicate closer agreement with the corresponding published reference, whereas values above one indicate a numerically more favorable value for that individual metric.

Numerically, the final checkpoint achieved a pulse-count accuracy 0.18 percentage points higher than the published reference value. However, it did not reach the published reference values for the reported waveform- and component-level quantities. In particular, its timing RMSE remained above the published value of 0.710 sampling periods, and its second-component-amplitude RMSE was approximately 6.9 times the published reference value of 0.0560. Because the local continuation and published reference originate from different training histories, these comparisons are descriptive and should not be interpreted as a paired statistical test. Nevertheless, the contrast between pulse-count accuracy and amplitude RMSE demonstrates that classification performance alone does not characterize component-level reconstruction fidelity.

### 5.2. Configuration Dependence of Component-Amplitude Reconstruction Error

To identify the pulse configurations under which component-amplitude recovery becomes difficult, we stratified the semi-synthetic evaluation set according to temporal separation, component-amplitude ratio, absolute component amplitude, and overall signal magnitude.

The resulting analysis shows that amplitude-reconstruction error is strongly configuration dependent. In particular, strongly imbalanced component amplitudes and large component magnitudes produce the largest errors, whereas the dependence on temporal separation is comparatively weaker. The Stage 2 model exhibits the same qualitative pattern, indicating that the amplitude-focused continuation does not remove the principal failure regime.

These observations characterize empirical failure modes within the present semi-synthetic protocol and should not be interpreted as a formal demonstration of parameter non-identifiability.

### 5.3. Calibration Transfer Under Controlled Distribution Shift

Under the five principal controlled distribution-shift conditions, the Stage 2 checkpoint achieved an aggregate pulse-count accuracy of 60.72%, and the corresponding calibration results are summarized in Table 7. Because scalar temperature scaling preserves the ordering of the logits, calibration did not alter the predicted class or the classification accuracy.

The uncalibrated aggregate ECE and NLL were 0.369567 and 4.542492, respectively. Applying the temperature fitted only on the nominal validation distribution produced an ECE of 0.370217 and an NLL of 4.667938. Thus, the nominally fitted temperature did not improve calibration after transfer to the tested shifted distributions.

In contrast, using the temperature fitted on validation data under the reference combined perturbation reduced the aggregate ECE to 0.114180 and the NLL to 0.636240. This result shows that the shifted logits can be substantially recalibrated when representative shifted validation data are available. However, this shifted validation setting is a sensitivity analysis and should not be interpreted as zero-shot calibration transfer.

Table 8 further reports the condition-level calibration results. The nominally fitted temperature produced little change in ECE under the tested shifts, whereas the shifted validation temperature substantially reduced ECE across all five conditions.

The condition-level results show the same overall pattern as the aggregate analysis. Calibration using the nominal validation temperature provides little or no improvement after transfer to the shifted distributions. By contrast, calibration using representative shifted validation data substantially reduces ECE. These results indicate that the effectiveness of post hoc temperature scaling depends on the relationship between the calibration and evaluation distributions.

### 5.4. Selective Risk and Coverage

Table 9 summarizes the Stage 2 risk–coverage behavior under the unified evaluator. On the clean evaluation set, the model achieved an accuracy of 99.12% with an AURC of 0.000860 and an E-AURC of 0.000821. Under the reference combined perturbation, accuracy decreased to 55.96%, while AURC and E-AURC increased to 0.336650 and 0.221088, respectively. These results indicate a substantial degradation of confidence-based risk ranking under the shifted input distribution.

The threshold-dependent analysis leads to the same conclusion. On the clean Stage 2 evaluation set, ${\mathrm{OER}}_{\mathrm{cond}}$ decreased from 0.008768 at ρ=0.50 to 0.004933 at the reference threshold ρ=0.85 and to $6.971\times 10^{-5}$ at ρ=0.9999. The corresponding coverage decreased from 1.000000 to 0.981920 and 0.448259, respectively. Thus, lower conditional OER at more stringent thresholds is accompanied by reduced coverage and should not be interpreted independently of the retained prediction fraction.

Under the reference combined perturbation, ${\mathrm{OER}}_{\mathrm{cond}}\left(0.85\right)$ was 0.435561 compared with 0.004933 on the clean evaluation set. The value of ρ=0.85 is therefore retained only as a reference diagnostic point rather than a universally preferred threshold.

For this binary classifier, scalar temperature scaling preserves the ordering of the class-confidence margin and therefore does not change the predicted class. Risk–coverage and threshold-dependent OER should consequently be interpreted as confidence diagnostics rather than evidence of improved pulse reconstruction. The observed risk–coverage behavior should consequently be attributed to the ranking information in the original model confidence rather than an improvement in ranking produced by temperature scaling. Calibration changes the numerical interpretation of confidence and the number of predictions exceeding a fixed confidence threshold, but it does not create the selective ordering reported in Table 9.

The 50% operating point is an empirical diagnostic rather than a prescribed acceptance threshold. It does not imply that half of the measured traces can be discarded without affecting a spectroscopy analysis. It also does not provide a formal bound on future error rates or a conformal coverage guarantee. The result is restricted to the evaluated checkpoint, perturbation protocol, and confidence-ranking rule.

## 6. Discussion

The principal finding of this study is not a uniform improvement across all pulse-analysis quantities but a separation among pulse-count classification, waveform reconstruction, component-parameter estimation, and confidence reliability. Under the nominal SAEFit protocol, continued training and configuration adjustment reduced timing RMSE from 2.177 to 1.308 sampling periods and increased double-pulse ASNR from 20.62 to 21.17 dB. Pulse-count accuracy remained high and reached 99.81%. In contrast, the reported second-component-amplitude RMSE changed only from 0.3869 to 0.3859 and remained substantially above the published reference value of 0.0560. Under the five principal controlled distribution-shift conditions, the Stage 2 checkpoint achieved an aggregate accuracy of 60.72%. Temperature fitted on the nominal validation distribution did not improve shifted ECE or NLL, whereas condition-matched fitting reduced ECE from 0.3696 to 0.1142 and NLL from 4.5425 to 0.6362.

### 6.1. Reconstruction Fidelity and Component-Parameter Recovery

The parallel improvement in timing RMSE and ASNR indicates that the continued checkpoints produced closer temporal alignment and waveform agreement under the semi-synthetic evaluation protocol. However, the absence of a comparable reduction in amplitude RMSE shows that improved waveform agreement did not translate directly into accurate component-amplitude recovery. This distinction is important because timing, waveform reconstruction, and amplitude estimation are generated by related but non-equivalent objectives.

Several factors could contribute to this behavior. The amplitude-related loss may provide a weaker optimization signal than the timing and classification objectives, the parameter head may not adequately represent the ambiguity between overlapping components, or the semi-synthetic training distribution may insufficiently constrain amplitude combinations that produce similar summed waveforms. Correlation between component timing, amplitude, and pulse shape may also lead to multiple parameter combinations with similar reconstruction errors. The present experiments do not distinguish among these explanations. The results therefore indicate an amplitude-specific limitation within the tested architecture and configuration range, but they do not constitute a formal demonstration of non-identifiability.

The contrast between classification and amplitude estimation is particularly informative. Distinguishing a single pulse from an overlapped pulse is a global waveform decision, whereas recovering the amplitude of each constituent requires a more detailed decomposition of the observed signal. A model may consequently achieve near-saturated pulse-count accuracy while retaining substantial component-level error. The final checkpoint achieved near-saturated pulse-count accuracy, including a slightly higher value than the published reference, while its component-amplitude error remained substantially above the published amplitude reference. Pulse-count accuracy should therefore not be used as a surrogate for timing or energy-related component fidelity. This limitation is especially relevant in radiation spectroscopy because the recovered pulse amplitude is closely associated with deposited-energy estimation. Consequently, a model that correctly identifies the number of overlapping events may still produce biased or unreliable energy-related component estimates. Pulse-count accuracy and waveform-level ASNR should therefore not be used as surrogates for component-amplitude fidelity.

The comparisons with the published 750-epoch values must nevertheless be interpreted cautiously. The published model, the local epoch-125 reproduction, and the continued checkpoints were obtained from different training histories. The published values provide a useful external reference, but the comparison is not equivalent to a controlled paired experiment. The present results support statements about improvement relative to the locally continued checkpoint; they do not establish general superiority over the original SAEFit model.

### 6.2. Confidence Reliability Under Controlled Distribution Shift

The revised calibration analysis shows that post hoc calibration is itself distribution dependent. Across the five principal controlled distribution-shift conditions, the Stage 2 checkpoint achieved an aggregate accuracy of 60.72%. A temperature fitted on the nominal validation distribution did not improve shifted calibration: aggregate ECE changed from 0.369567 to 0.370217 and NLL from 4.542492 to 4.667938.

By contrast, fitting the temperature using validation data under the reference combined perturbation reduced aggregate ECE to 0.114180 and NLL to 0.636240. This result demonstrates that the shifted logits can be recalibrated when representative shifted validation data are available, but it does not establish zero-shot transfer of a calibration parameter estimated under nominal conditions.

The OER analysis leads to a complementary conclusion. The measured overconfident-error rate depends on the selected confidence threshold and the input distribution. On clean Stage 2 data, OER_cond_ decreased substantially as the threshold became more stringent, but this decrease was accompanied by reduced coverage. Under the reference combined perturbation, conditional OER remained high over the same threshold range. Accordingly, ρ=0.85 is used only as a reference diagnostic point rather than a universally preferred operating threshold.

These results distinguish calibration from robustness. Temperature scaling changes the numerical interpretation of class confidence but does not recover incorrect class decisions, improve component reconstruction, or guarantee calibration transfer to a different input distribution.

### 6.3. Role of Semi-Synthetic Supervision

The semi-synthetic construction provides an important but limited source of component-level supervision. Because each double pulse is formed by shifting, scaling, and summing measured single-pulse traces, the constituent timing and amplitude parameters are known by construction. The component waveforms also retain signal characteristics derived from detector measurements, which makes this protocol more representative than one based entirely on analytically generated pulse shapes.

Nevertheless, semi-synthetic overlaps are not equivalent to independently labeled measured double pulses. Their construction may not reproduce all the dependencies among count rate, detector response, pulse shape, electronics behavior, baseline evolution, saturation, and sampling. It may also omit correlations that arise when naturally occurring events overlap under changing experimental conditions. The component-level RMSE obtained from this protocol therefore characterizes recovery under the specified construction rules.

The measured double-pulse traces provide evidence of naturally occurring waveform overlap but do not contain independently verified labels for each component. They cannot support supervised timing or amplitude RMSE without additional assumptions. For this reason, the present study does not transfer the observed semi-synthetic amplitude error directly to measured double pulses. Conversely, the absence of component labels should not be addressed through pseudo-labels derived from the model being evaluated because doing so would make the reference dependent on the prediction.

The separation between semi-synthetic reconstruction evaluation and perturbed classification evaluation is therefore methodologically necessary. The former addresses component recovery when labels are available by construction, whereas the latter addresses confidence behavior under specified input changes. Neither experiment alone establishes accurate decomposition of naturally overlapped pulses under changing detector conditions.

### 6.4. Limitations and Validity

Several limitations define the scope of the conclusions. First, the continuation study used a bounded configuration search involving existing learning rates and loss weights. The limited change in amplitude RMSE suggests that the tested controls were insufficient, but it does not exclude improvement through a broader search, an alternative loss formulation, or a different architecture.

Second, the repeated-run analysis used five independent random seeds and therefore provides an empirical estimate of training variability. The paired results support systematic improvements in pulse-count accuracy and timing RMSE but not component-amplitude RMSE. Nevertheless, five runs do not fully characterize the distribution of training outcomes or sensitivity to all the implementation choices.

Third, model and checkpoint selection used feedback from the public evaluation protocol. The resulting checkpoint values should be interpreted as optimized outcomes within that protocol rather than untouched estimates of performance on an independent detector population. Evaluation using an external detector run or a prospectively held-out dataset would provide a stronger test of generalization.

Fourth, the distribution-shift analysis was based on controlled input-level perturbations and transformations. In addition to noise and baseline changes, the revised experiments include multiplicative gain variation and parametric pulse-shape variation. These additional experiments broaden the controlled stress-test space but do not constitute validation on an independent detector population or naturally overlapped events with independently verified component labels. Other detector-condition changes, including nonlinear electronics response, saturation, altered sampling behavior, and changes in noise spectra or event statistics, remain outside the present evaluation scope.

Fifth, the calibration temperature was estimated using validation data drawn from the defined perturbation protocol. Its effectiveness under a qualitatively different or previously unseen shift remains unknown. Temperature scaling is also a global scalar correction and cannot represent input-dependent or class-dependent calibration errors.

Finally, the selective-prediction experiment provides an empirical risk–coverage relationship rather than a formal guarantee. It does not establish that a selected coverage will maintain the same error rate in a future detector run. The analysis also does not quantify the physical or operational consequences of deferring low-confidence events.

### 6.5. Future Directions

The most immediate methodological priority is component-amplitude recovery. Future work should examine amplitude-specific objectives, parameterizations that explicitly preserve consistency between the reconstructed waveform and estimated component amplitudes and constraints based on pulse area or other energy-related quantities. These alternatives should be evaluated under the same component-labeled protocol and across repeated training runs to determine whether improvements are systematic.

A more decisive evaluation requires measured double pulses with independently established component timing and amplitude. Such labels might be obtained through controlled pulse injection, independently time-referenced event generation, or another experimental design in which the constituent signals are known before overlap. This would permit direct assessment of whether performance observed on semi-synthetic overlaps transfers to measured pile-up events.

Future evaluation should move beyond the present controlled gain and parametric pulse-shape transformations toward independently acquired detector runs that capture realistic changes in baseline dynamics, noise spectra, nonlinear electronics response, saturation, sampling, count rate, and other operating-condition effects. Evaluation across independent acquisition runs or detector systems would help to distinguish calibration that is specific to one stress construction from calibration that transfers across operating conditions.

More expressive uncertainty methods, including deep ensembles, may improve uncertainty representation but require repeated training and substantially greater computational cost [22]. Conformal methods could provide complementary coverage statements if their assumptions and calibration design are satisfied [39]. Neither approach should be assumed to correct weak amplitude decomposition; reconstruction quality and confidence reliability would still require separate evaluation.

Finally, a hybrid analysis path combining fast learned inference with nonlinear fitting for selected traces should be assessed at the workflow level. Such an assessment should report not only selective classification accuracy but also processing time, deferred-trace frequency, component-reconstruction error, and the effect on downstream spectroscopic quantities. This would determine whether confidence-based selection provides a measurable scientific benefit beyond improved calibration metrics.

## 7. Conclusions

This study examined whether reconstruction fidelity and confidence reliability improve concurrently in overlapped radiation-detector pulse analysis. Under the nominal SAEFit protocol, continued training reduced timing RMSE from 2.177 to 1.308 sampling periods and increased double-pulse ASNR from 20.62 to 21.17 dB, while pulse-count accuracy reached 99.81%. These improvements were not accompanied by comparable component-amplitude recovery, and the repeated-run analysis did not provide statistical evidence for improved amplitude RMSE.

Confidence calibration exhibited a different limitation under controlled distribution shift. Across the five principal shift conditions, the Stage 2 model achieved an aggregate accuracy of 60.72%. A temperature fitted on the nominal validation distribution did not improve shifted calibration, whereas condition-matched fitting reduced aggregate ECE from 0.369567 to 0.114180 and NLL from 4.542492 to 0.636240. The OER analysis further showed that high-confidence error rates depend on the selected confidence threshold and should therefore be interpreted together with coverage.

These findings support a clear distinction among pulse-count classification, component-level reconstruction, and confidence reliability. High classification accuracy does not establish accurate recovery of constituent amplitudes, and improved calibration does not correct classification or decomposition errors. The present evidence is restricted to the evaluated semi-synthetic reconstruction protocol and controlled input-distribution shifts. Further progress requires independently labeled measured overlaps, amplitude-specific modeling, and validation across independent detector conditions.

## Figures and Tables

**Table 1:** Data sources, available reference information, and supported evaluations.

Data Category	Waveform Origin	Available Reference	Supported Evaluation
Measured single pulses	Detector measurements	Single-pulse class and measured waveform	Pulse-count classification and single-pulse reconstruction
Semi-synthetic double pulses	Transformed and summed measured single pulses	Component timing and amplitude defined by construction	Double-pulse classification, waveform reconstruction, and component-level errors
Measured double pulses	Detector measurements	Double-pulse waveform without independent component labels	Evidence of naturally occurring overlap; no supervised component-level RMSE because independent constituent labels are unavailable
Perturbed evaluation traces	Controlled perturbations applied to evaluation waveforms	Retained pulse-count labels	Shifted classification, calibration, and selective-risk assessment

**Table 2:** Selected configurations for the two continuation stages.

Stage	Initial Checkpoint	τ	$\tau_{c}$	λ	Learning Rate	Primary Evaluation Quantity
Timing-focused	Continued checkpoint near epoch 500	5.0	0.05	0.01	$3\times 10^{-4}$	Timing RMSE
Amplitude-focused	Selected timing-focused checkpoint	3.0	0.05	0.05	$2\times 10^{-4}$	Amplitude RMSE

**Table 3:** Parameters of the five primary controlled distribution-shift conditions. All values are expressed in normalized waveform units.

Condition	Noise σ	Baseline Offset
Noise only	0.05	0
Baseline only	0	U(−0.10,0.10)
Reference combined	0.05	U(−0.10,0.10)
Mild combined	0.03	U(−0.05,0.05)
Strong combined	0.08	U(−0.15,0.15)

**Table 4:** Performance under the nominal SAEFit evaluation protocol.

Checkpoint	Acc. (%)	${\mathrm{ASNR}}_{s}$	${\mathrm{ASNR}}_{p1}$	${\mathrm{ASNR}}_{p2}$	${\mathrm{ASNR}}_{d}$	${\mathrm{RMSE}}_{t}$	${\mathrm{RMSE}}_{a,2}$
Epoch 125	99.40	21.12	16.76	16.89	20.06	–	–
Published 750-epoch reference	99.63	21.57	20.20	20.32	21.94	0.710	0.0560
Continued checkpoint	99.77	21.16	18.59	18.16	20.62	2.177	0.3869
Timing-focused checkpoint	99.80	21.29	19.17	18.79	21.10	1.402	0.3866
Amplitude-focused checkpoint	99.81	– ^a^	19.27	18.80	21.17	1.308	0.3859

^a^ The single-pulse ASNR for the amplitude-focused checkpoint was not retained in the archived nominal-protocol evaluation output. The supplementary component-level reevaluation used semi-synthetic double pulses and therefore does not provide a directly comparable ASNR_*s*_ value.

**Table 5:** Complete component-amplitude reevaluation of the two final continuation stages on 112,000 semi-synthetic double pulses. RMSE_*a*,joint_ summarizes the errors of both ordered amplitude components.

Checkpoint	${\mathrm{RMSE}}_{a,1}$	${\mathrm{RMSE}}_{a,2}$	${\mathrm{RMSE}}_{a,\mathrm{joint}}$
Timing-focused checkpoint	1.5511	0.3807	1.1293
Amplitude-focused checkpoint	1.5518	0.3807	1.1298

**Table 6:** Repeated-run analysis over five independent random seeds. Values for each stage are reported as mean ± standard deviation. Differences and 95% confidence intervals are calculated for paired Stage 2–Stage 1 comparisons.

Metric	Stage 1	Stage 2	Difference	95% CI
Accuracy (%)	98.85±0.1250	99.21±0.0906	+0.3565	[0.2355,0.4776]
Timing RMSE	2.4776±0.1773	2.1849±0.2196	−0.2926	[−0.4773,−0.1079]
${\mathrm{RMSE}}_{a,1}$	0.4147±0.0201	0.3992±0.0065	−0.0154	[−0.0336,0.0027]
${\mathrm{RMSE}}_{a,2}$	0.4239±0.0527	0.3987±0.0060	−0.0252	[−0.0927,0.0423]

**Table 7:** Aggregate Stage 2 calibration results over the five principal controlled distribution-shift conditions. The nominal validation temperature was fitted using nominal validation data, whereas the shifted validation temperature was fitted using validation data under the reference combined perturbation.

Probability Output	Accuracy (%)	ECE	NLL	E-AURC
Uncalibrated	60.72	0.369567	4.542492	0.178948
Nominal validation temperature	60.72	0.370217	4.667938	0.174223
Shifted validation temperature	60.72	0.114180	0.636240	0.160801

**Table 8:** Stage 2 calibration error under the five principal controlled distribution-shift conditions. Clean-fit denotes temperature fitted on nominal validation data; shifted-fit denotes temperature fitted under the reference combined validation perturbation.

Condition	Accuracy (%)	${\mathrm{ECE}}_{\mathrm{uncal}}$	${\mathrm{ECE}}_{\mathrm{clean}}$	${\mathrm{ECE}}_{\mathrm{shift}}$
Noise only	71.06	0.259625	0.260442	0.110801
Baseline only	57.94	0.400734	0.401307	0.129687
Reference combined	55.96	0.417935	0.418567	0.100667
Mild combined	65.57	0.316608	0.317379	0.120346
Strong combined	53.09	0.452936	0.453390	0.109397

**Table 9:** Stage 2 risk–coverage summary under the unified evaluator.

Evaluation Condition	Accuracy (%)	AURC	E-AURC
Clean	99.12	0.000860	0.000821
Reference combined	55.96	0.336650	0.221088

## Data Availability

The waveform data and baseline SAEFit implementation are based on the public SAEFit release v1.0.0 (https://github.com/qloongx/SAEFit.git, accessed on 9 October 2026). Experimental settings and evaluation procedures are provided in the manuscript. Additional scripts, checkpoints, and result files are available from the corresponding author upon reasonable request.
